# Depicting the Discrepancy between *Tri* Genotype and Chemotype on the Basis of Strain CBS 139514 from a Field Population of *F. graminearum* Sensu Stricto from Argentina

**DOI:** 10.3390/toxins8110330

**Published:** 2016-11-12

**Authors:** Tomasz Kulik, Maciej Buśko, Katarzyna Bilska, Anna Ostrowska-Kołodziejczak, Anne D. van Diepeningen, Juliusz Perkowski, Sebastian Stenglein

**Affiliations:** 1 Department of Botany and Nature Protection, University of Warmia and Mazury in Olsztyn, Plac Łódzki 1, Olsztyn 10-727, Poland; tomaszkulik76@gmail.com; 2 Department of Chemistry, Poznań University of Life Sciences, ul. Wojska Polskiego 75, Poznań 60-625, Poland; mabu@au.poznan.pl (M.B.); ostrowska.anna.maria@gmail.com (A.O.-K.); julperk@au.poznan.pl (J.P.); 3 CBS-KNAW Fungal Biodiversity Centre, Uppsalalaan 8, Utrecht 3584 CT, The Netherlands; a.diepeningen@cbs.knaw.nl; 4 Institute of Biodiversity and Ecosystem Dynamics, University of Amsterdam, Science Park 904, Amsterdam 1098 XH, The Netherlands; 5 Laboratorio de Biología Funcional y Biotecnología (BIOLAB)-Comisión de Investigaciones Científicas de la provincia de Buenos Aires-Instituto de Investigaciones en Biodiversidad y Biotecnología-Consejo Nacional de Investigaciones Científicas y Técnicas (CICBA-INBIOTEC, CONICET), Av. República de Italia 780, Azul 7300, Buenos Aires, Argentina; stenglein@faa.unicen.edu.ar; 6 Cátedra de Microbiología-Facultad de Agronomía de Azul-UNCPBA, Av. República de Italia 780, Azul 7300, Buenos Aires, Argentina

**Keywords:** *Fusarium*, *Tri* genotypes, chemotypes

## Abstract

Recent studies on a field population of *F. graminearum* sensu stricto from Argentina revealed an atypical panel of strains identified through PCR genotyping as 15ADON genotypes, but producing high levels of 3ADON. Based on representative strain CBS 139514, we asked if the discrepancy between the trichothecene genotype and chemotype might result from an inter-chemotype recombination of the chemotype-determining genes. To answer this, we sequenced the complete core *Tri* gene cluster (around 30,200 bp) from this strain and compared its sequence to sequence data of typical type B trichothecene genotypes/chemotypes. Sequence alignment showed that CBS 139514 has an identical sequence within the entire core *Tri* cluster to the 15ADON genotype. The revealed discrepancy underlines the need for using both molecular and chemical methods for reliable characterization of toxigenic strains of *Fusarium*.

## 1. Introduction

Trichothecenes produced by the phytopathogenic *Fusarium* species contribute to the ability of fungi to cause plant diseases and induce toxicoses in animals and humans. These mycotoxins can be grouped into two classes based on the presence (type B trichothecenes) versus absence (type A trichothecenes) of a keto group at the C-8 position. Type B trichothecenes are of the greatest concern in barley and wheat-growing regions worldwide and include deoxynivalenol (DON), nivalenol (NIV), and their acetylated derivatives: 3-acetyldeoxynivalenol (3ADON), 15-acetyldeoxynivalenol (15ADON), and 4-acetylnivalenol (4ANIV, syn. fusarenone-X) [1,2]. Each trichothecene compound has various and multiple effects on eukaryote cells, including inhibition of proteins, DNA and RNA synthesis, inhibition of mitochondrial function and effects on cell division and membrane effects. In animal cells, they induce apoptosis, a programmed cell death response. Acetyl derivatives of DON are more toxic to cereals than NIV, although NIV has been found to be more toxic to mammalian cells [1]. Many toxigenic *Fusarium* species can contaminate the grains with these mycotoxins; however, *F. graminearum* sensu stricto (s.s.) is nowadays the most frequently isolated species worldwide. *F. graminearum* s.s. can belong to different chemotypes: 3ADON (producing DON and 3ADON), 15ADON (producing DON and 15ADON) and NIV (producing NIV and 4ANIV) [3,4].

Determination of chemotype distribution and composition has been the subject of extensive studies in geographically diverse fungal populations [4]. Such information is imperative in order to determine population structure, monitor chemotype shifts and predict contamination of grains with certain trichothecene compounds [5,6,7]. Chemotypes can be determined using a chemical method, based on which toxin accumulates in axenic cultures [8]. It includes the isolation of fungi from infected spikes and the determination of the chemotypes on the isolates using either liquid or gas chromatography, often coupled to mass spectrometry.

Sequencing of genes involved in trichothecene biosynthesis (*Tri* genes) enabled the determination of the genetic basis of chemotype-specific differences and the discovery of a correlation between the polymorphism of some *Tri* genes, i.e., *Tri3*, *Tri12* and *Tri13*, and chemotypes [3], initiated the development of numerous molecular markers for quick and reliable chemotype prediction. These molecular methods are mainly based on specific sets of primers and/or probes [9,10,11,12], which enable the analysis of fungal populations by screening a large set of isolates in a chemotype diversity context far beyond that provided by chemical methods in terms of speed and simplicity [4].

However, it should be noted that the translation of genotype into chemotype is complicated by gene interactions that lead to tremendous strain-dependent chemical diversity in *Fusarium* [13]. This is observed as remarkable, strain-dependent variation in levels of toxin production [14] and the simultaneous production of considerable amounts of compounds characteristic of other chemotypes [5]. Recently, studies on a field population of *F. graminearum* sensu stricto from Argentina revealed an atypical panel of strains showing a discrepancy between the *Tri* genotype and chemotype [15].

Based on CBS 139514 from this group of strains, we asked if the discrepancy between the *Tri* genotype (identified as 15ADON using PCR assay) and chemotype (determined as 3ADON chemotype using chemical analysis) might result from inter-chemotype recombination involving the chemotype-determining genes. To answer this, we sequenced the complete core *Tri* cluster of this strain and compared its sequence to the *Tri* sequence data of the other three type B *Tri* genotypes/chemotypes. We show that production of high quantities of 3ADON by the atypical strain CSB 139514 cannot be predicted by a polymorphism within the core *Tri* cluster. The revealed discrepancy between the *Tri* genotype and chemotype underlines the need for using both molecular and chemical methods for reliable characterization of toxigenic *Fusarium* spp.

## 2. Results

### 2.1. Molecular Analysis

We sequenced, assembled and annotated the complete core *Tri* cluster gene from the strain CBS 139514. For comparison, our sequencing analysis also incorporated five strains representing the other *Tri* genotypes/chemotypes within *F. graminearum* s.s. (Table 1). The complete sequences of the sequence data have been deposited in the NCBI database under the GenBank accession numbers given in Table 1. The identified genotypes were in agreement with the chemotypes, except for strain CBS 139514. BLASTN analysis of the complete core *Tri* cluster (around 30,200 bp) showed that CBS 139514 was identical within the entire cluster to strain CBS 139513, originating from the same collection site and identified as the 15ADON genotype/chemotype (Table 1). The obtained result indicates that high 3ADON production by CBS 139514 cannot be explained by inter-chemotype recombination or hybridization within the core *Tri* cluster. In this study, we also obtained the complete sequences of the *Tri101* gene from the studied strains. The *Tri101* gene encodes a trichothecene 3-*O*-acetyltransferase that can convert DON into 3ADON [16] and is not located near the *Tri* core cluster but between the phosphate permease gene and the UTP-ammonia ligase gene [17]. Sequence analysis (1356 bp) showed that the *Tri101* gene displays a high level of conservation within the studied group of strains. CBS 139514 differs by only one synonymous SNP (T/C, 606 bp) from CBS 139513 (15ADON genotype/chemotype) and displays 100% identity to CBS 138562 (3ADON genotype/chemotype).

### 2.2. Chemotype Determination of Fungal Strains

Table 1 shows detailed data on toxin production by the examined strains. Results of our chemical analysis confirmed the highest production of 3ADON by strain CBS 139514. All tested strains co-produced trichothecene compounds in different quantities, which is in agreement with the previous studies by Mugrabi de Kuppler et al. [5] and Castañares et al. [15].

## 3. Discussion

It has been previously demonstrated that the sequence polymorphism within the *Tri3* and *Tri12* genes can predict DON chemotypes [10,11,12]. Further functional analyses of the *Tri8* enzyme (*Tri8*) revealed that its differential activity determines the 3ADON and 15ADON chemotypes in *Fusarium* [20]. *Tri8* from 3ADON strains catalyzes the deacetylation of the trichothecene biosynthetic intermediate 3,15-diacetyldeoxynivalenol at carbon 15 to yield 3ADON, whereas *Tri8* from 15ADON strains catalyzes the deacetylation of 3,15-diacetyldeoxynivalenol at carbon 3 to yield 15ADON [20]. In addition, chemotypes differ in the expression levels of *Tri* genes. The comparison of gene expression among the three chemotypes showed that relative expression of *Tri* genes was higher in 3ADON-producing strains compared with 15ADON and NIV strains, which contribute to the higher levels of DON produced by 3ADON strains in infected grains [21].

Previous studies showed that the discrepancies between molecular and chemical analyses result from either inter-chemotype recombination involving the chemotype-determining genes [22] or false signals of genotyping assays [13,23]. In this study, we depicted the discrepancy between the *Tri* genotype and chemotype of CBS 139514 by showing that the sequence of this strain within the complete core *Tri* cluster is identical to the strain of the 15ADON genotype/chemotype (CBS 139513). We speculate that 3ADON production by this strain may result from differences in enzyme activity leading to the conversion of DON to 3ADON. In *F. graminearum*, *Tri101* more effectively accommodates DON than *Tri101* from *F. sporotrichioides* [16]. One could speculate whether the high accumulation of 3ADON by CBS 139514 may result from the increased activity of *Tri101*.

It would be interesting to know whether small changes within the *Tri101* gene or its expression in *F. graminearum* s.s. might affect the differential activity of *Tri101* leading to the differences in 3ADON accumulation in axenic media. Overall, our results of this study have important implications in further toxicological studies on *Fusarium* spp. The revealed discrepancy between the *Tri* genotype and chemotype underlines the need for using both molecular and chemical methods in studies of toxigenic fungi. Molecular characterization of individual strains based on DNA sequencing or PCR genotyping allows for describing and quantifying fungal diversity. Identification and quantification of *Tri* genotypes can be used to predict toxigenic chemotypes; however, molecular approaches should be validated by chemical analysis of individuals that represent the allelic diversity of the target gene in the population [24]. Linking molecular identification with chemical characterization will allow the detection of other atypical strains, which may have potential implications for a better understanding of chemical diversity in *Fusarium*. However, this requires a reference database containing a satisfactory taxonomic sampling of sequences, together with chemical data. Our next study will aim to characterize a more comprehensive panel of field isolates from various geographic locations. A new reference database (ToxGen) containing both molecular and chemical data will be soon open on the PlutoF platform [25,26], which allows the rapid submission, retrieval, and analysis of study, specimen and sequence data [27]. The main purpose of creating such a database is to provide a joint corpus of molecular and toxicological metadata which could be used by anyone seeking to identify and characterize *Tri* genotypes using sequence data. We believe that a deeper understanding of fungal biodiversity in agroecosystems will only be possible by the joint collaboration of researchers working in the fields of fungal taxonomy, epidemiology and mycotoxicology.

## 4. Materials and Methods

### 4.1. Fungal Strains and Growth Conditions

Strain CBS 139514 was selected from an atypical panel of strains of *F. graminearum* s.s. from Argentina identified using PCR assay as 15ADON genotypes but producing the highest levels of 3ADON [15]. For comparison, we additionally subjected to both molecular and chemical analysis five other strains: CBS 138562, CBS 119173, CBS 138561, CBS 139513, and CBS 138563. All strains have been maintained in the CBS–Fungal Biodiversity Centre, Utrecht, The Netherlands. A detailed description of the fungal strains is given in Table 1. For DNA extraction, fungal strains were incubated on Petri plates (Ø 80 mm) with PDA medium at 24 °C in the dark for seven days. For chemical analysis, fungal strains using two biological replicates were incubated on YES medium at 27 °C in the dark for 14 days.

### 4.2. Molecular Analysis

#### 4.2.1. DNA Isolation and DNA Sequencing

First 0.1 g of mycelium scraped from the surface of PDA plates was used for DNA extraction from fungi using a ChargeSwitch*^®^* gDNA Plant Kit (Invitrogen, Carlsbad, CA, USA) following the manufacturer’s recommendations.

Whole genome libraries were prepared using the Nextera XT kit (Illumina, San Diego, CA, USA) from genomic DNA extracted from mycelium. Constructed libraries were sequenced on the Illumina Miseq platform with the 250 bp paired-end read, version 2 (Illumina, San Diego, CA, USA). The fungal genomes were sequenced in a multiplexed format (six to seven samples per run), where an oligonucleotide index barcode was embedded within adapter sequences that were ligated to DNA fragments.

#### 4.2.2. Assembly, Annotation of *Tri* Genes and Sequence Analyses

For assembling complete core *Tri* clusters, reads were aligned to the core *Tri* clusters of *F. graminearum* s.s. strain GZ3639 and *F. dactylidis* strain NRRL 29380 (KP057243). For assembling the complete *Tri101* gene, reads were aligned to the *F. graminearum Tri101* gene (FJ595991). Sequence reads were aligned with Geneious (v.6.1.6 created by Biomatters, Auckland, New Zealand) [28] with custom sensitivity settings to allow gaps with a maximum of 20% per read, minimum overlap of 100 or 200 bp, identity of 99%, a word length of 18 with words repeated more than 12 times ignored, a maximum of 20% mismatches per read, an index word length of 13, and a maximum ambiguity of 4. We mapped multiple best matches randomly using fine-tuning iteration up to 100 times. Next, sequence reads were assembled to conserved sequences revealed by alignment. Resulting contigs were iteratively extended through recruiting sequence data by aligning to contig edges until no gaps remained. Overlapping contigs were assembled de novo into a single scaffold. This scaffold was inspected for complete coverage by alignment of sequence reads with custom sensitivity settings as described above. Finally, the resulting scaffolds were trimmed and rotated to facilitate comparative analysis. Annotations were performed using Geneious software based on *Tri* sequence data deposited under accession numbers: AF359361, KP057243 and FJ595991. The strains were assigned to particular genotypes, either 3ADON, 15ADON or NIV, using BLASTN comparisons of complete sequences of *Tri12* gene.

### 4.3. Chemotype Determination by GC-MS

*Tri* chemotypes of the strains incubated on YES medium were determined by GC-MS analysis as previously described by Perkowski et al. [19]. An additional chemical analysis using sterile rice substrate was performed for the strain CBS 139514 using the method described in Castañares et al. [15]. The strains were assigned to particular chemotypes, either 3ADON, 15ADON or NIV, after the toxin detected in the media at highest concentration.

## Figures and Tables

**Table 1:** List of fungal strains used in this study.

CBS Strain	Genotype/Chemotype	*GenBank Accession Number* (Core *Tri* Cluster/*Tri101*)	Trichothecene Production mg·kg^−1^					Chemical Analyses
			DON	3ADON	15ADON	NIV	4ANIV	
139514 ^1^	15ADON/3ADON	KU572431/KX774500	17.9	7.15	1.3	n.d.	n.t.	[15]
			0.59 ± 0.03	0.68 ± 0.01	0.03 ± 0.002	0.01 ± 0.001	0.02 ± 0.001	this study ^3^
			25.42 ± 0.96	273.12 ± 30.4	0.43 ± 0.01	n.d.	n.t.	this study ^4^
139513 ^2^	15ADON/15ADON	KU572432/KX774499	4.08	n.d.	2.04	n.d.	n.t.	[15]
138561	15ADON/15ADON	KU572429/KX774496	2.52 ± 0.04	0.46 ± 0.06	1.37 ± 0.02	n.d.	n.d.	[18]
			2.16 ± 0.94	1.42 ± 0.3	1.6 ± 0.48	0.12 ± 0.02	0.96 ± 0.01	this study ^3^
138562	3ADON/3ADON	KU572434/KX774497	1.3 ± 0.02	3.75 ± 0.05	n.t.	n.t.	n.t.	[18]
			0.91 ± 0.42	2.48 ± 1.26	0.09 ± 0.02	0.03 ± 0.02	0.02 ± 0.01	this study ^3^
119173	3ADON/3ADON	KU572433/KX774495	5.5 ± 2.7	24.7 ± 1.1	0.03 ± 0.01	0.08 ± 0.05	0.02 ± 0.01	this study ^3^
138563	NIV/NIV	KU572430/KX774498	0.01 ± 0.01	0.04 ± 0.03	n.d.	1.86 ± 1.6	0.88 ± 0.43	this study ^3^

^1^ the strain was ascribed as 3–6 isolate in Castañares et al. [15]; ^2^ the strain was ascribed as 33–22 isolate in Castañares et al. [15]; n.t.—not tested; n.d.—not detected; ^3^ chemotypes determined by the method described by Perkowski et al. [19] using YES medium; ^4^ chemotype determined by the method described in Castañares et al. [15] using sterile rice substrate. The strains were assigned to particular genotypes, either 3ADON, 15ADON or NIV, using BLASTN comparisons of complete sequences of *Tri12* gene. The strains were assigned to particular chemotypes, either 3ADON, 15ADON or NIV, after the toxin was detected in YES media at highest concentration.
